# Gastrointestinal disorders in Parkinson’s disease and other Lewy body diseases

**DOI:** 10.1038/s41531-023-00511-2

**Published:** 2023-05-05

**Authors:** Masaaki Hirayama, Hiroshi Nishiwaki, Tomonari Hamaguchi, Kinji Ohno

**Affiliations:** 1 Department of Pathophysiological Laboratory Sciences, Nagoya University Graduate School of Medicine, Nagoya, Japan; 2 Division of Neurogenetics, Center for Neurological Diseases and Cancer, Nagoya University Graduate School of Medicine, Nagoya, Japan

**Keywords:** Parkinson's disease, Microbiology

## Abstract

Parkinson’s disease (PD) is pathologically characterized by the abnormal accumulation of α-synuclein fibrils (Lewy bodies) in the substantia nigra and other brain regions, although the role of Lewy bodies remains elusive. Constipation usually precedes the motor symptoms in PD, which is in accordance with the notion that α-synuclein fibrils start from the intestinal neural plexus and ascend to the brain in at least half of PD patients. The gut microbiota is likely to be involved in intestinal and brain pathologies. Analyses of the gut microbiota in PD, rapid-eye-movement sleep behavior disorder, and dementia with Lewy bodies suggest three pathological pathways. First, *Akkermansia*, which is increased in PD, degrades the intestinal mucus layer and increases intestinal permeability, which triggers inflammation and oxidative stress in the intestinal neural plexus. Second, decreased short-chain fatty acids (SCFAs)-producing bacteria in PD reduce the number of regulatory T cells. Third, SCFAs also aggravate microglial activation with an unelucidated pathway. In addition, in dementia with Lewy bodies (DLB), which is another form of α-synucleinopathies, increased genera, *Ruminococcus torques* and *Collinsella*, may mitigate neuroinflammation in the substantia nigra by increasing secondary bile acids. Interventions for the gut microbiota and their metabolites may potentially delay or mitigate the development and progression of PD and other Lewy body diseases.

## Introduction

Parkinson’s disease (PD) is pathologically characterized by abnormal accumulation of α-synuclein fibers (Lewy bodies) in the substantia nigra and other brain regions. However, the role of Lewy bodies has not been fully elucidated. PD is a movement disorder characterized by rigidity (muscle stiffness), postural instability, gait disturbance, bradykinesia (slowness of muscle movement), and resting tremor. However, PD patients often present with non-motor symptoms, including mild to severe cognitive impairment. Gastrointestinal symptoms of PD constitute the most common non-motor symptoms, with constipation being present in ~80% of PD patients^1^. Constipation usually precedes the motor symptoms of PD^2^ and worsens with disease progression. Similarly, the transit times of the small and large intestines are prolonged in PD^3^. In addition to constipation, non-motor symptoms such as rapid-eye-movement (REM) sleep behavior disorder (RBD), excessive daytime sleepiness, anosmia/hyposmia, and depression occur before the onset of the motor symptoms of PD.

Braak et al. examined the appearance of Lewy bodies in PD patients without cognitive symptoms and healthy participants and observed that the distribution of Lewy bodies ascended from the dorsal vagal nucleus to the locus coeruleus and substantia nigra^4^. The dorsal vagal nucleus innervates the gastrointestinal tract, and its distribution coincides with the manifestation of constipation as a prodromal PD symptom. In RBD, the sub-laterodorsal tegmental nucleus (SLD), located from the medulla oblongata to the bridge, is affected. SLD has a direct or indirect input to the spinal motor nuclei and inhibits skeletal muscle contraction during REM sleep, and its impairment may cause abnormal REM sleep behavior^5^. The locus coeruleus may also be involved in hypersomnolence in PD patients.

Two pathways have been proposed for the entry of a neurotropic pathogen into the nervous system in PD^6^. One pathway is from the olfactory bulb, and the other is from the oral and nasal cavities to the gastrointestinal tract and enteric nervous system (ENS)^6^. Although the olfactory pathway still remains controversial, the presence of the enteric pathway is supported by the ascending ɑ-synuclein pathology from the brainstem to the substantia nigra along with the ascendance of the disease loci for constipation, RBD, and depression prior to the onset of motor symptoms^7^. As discussed below, an approximately 50% reduction in the prevalence of PD after truncal vagotomy also supports the enteric pathway of PD^8,9^.

Radiological analysis has indicated the following two types of PD: body-first and brain-first PD^10^. PD patients with RBD, as well as idiopathic RBD patients, show the body-first trajectory, which represents an ascending progression from the intestinal tract and the cardiac sympathetic nervous system to the striatum. In contrast, PD patients without RBD show the brain-first trajectory, which represents descending progression from the amygdala to the intestinal tract and cardiac sympathetic nervous system. Gut microbiota is likely to be involved in the body-first PD.

In this article, we review the involvement of the gut–brain axis in the development and progression of PD from the perspective of the gut microbiota.

## Parkinson’s disease and gastrointestinal disorders

Inflammatory bowel diseases (IBD) include ulcerative colitis (UC) and Crohn’s disease (CD). A meta-analysis revealed that UC and CD increase the risk ratio of PD 1.28- and 1.30-fold, respectively^11^. Interestingly, anti-tumor necrosis factor (TNF) therapy for IBD reduces the risk of PD^12,13^. The increased risk of PD in IBD patients is consistent with the fact that increased intestinal permeability is causally related to PD. However, only one in 23 colectomized patients with UC (average age, 52.0 years) has shown the presence of Lewy bodies in the ENS^14^, which indicates a low prevalence of abnormal α-synuclein fibrils in the ENS in UC. A genome-wide association study has shown that the leucine-rich repeat kinase 2 (*LRRK2*) gene is a major locus associated with increased susceptibility to CD^15^. Pathogenic variants of *LRRK2* are the most common cause of familial PD and also account for ~1% of sporadic PD^16^. As high LRRK2 levels are observed in the inflamed colon in CD patients and in peripheral immune cells in sporadic PD patients, high levels and/or activities of LRRK2 may aggravate inflammatory processes in both CD and PD^17,18^. Thus, high LRRK2 levels in CD may be a potential biomarker for PD development and a potential therapeutic target to reduce the risk of PD in CD patients^17^.

Epidemiological studies show that total vagotomy reduces the hazard ratio of PD by approximately 50%^8,9^. The prion-like transmission of α-synuclein fibrils via the vagal nerve has also been observed in animal models^19^. Furthermore, stimulation of the vagal nerve in mice after administering lipopolysaccharides (LPS) suppresses the inflammation-induced production of TNF-α and interleukin (IL)-6 by microglia, which is not observed in vagotomized mice^20^. In addition, appendectomy may^21^ or may not^22^ reduce the risk of PD, which is in agreement with the observation that α-synuclein pathology begins in the vermiform appendix^23^. The vagal nerve is likely to play an essential role in the transmission of α-synuclein fibrils from the gut to the brain^24^.

Norovirus infection increases ɑ-synuclein production in the ENS, and ɑ-synuclein accelerates intestinal inflammation, suggesting an association between microbial infection and ɑ-synuclein pathology^25^. However, these observations are limited to PGP9.5-positive neurites, and the plexuses of Meissner and Auerbach have not been evaluated for the presence of α-synuclein-positive inclusions. Intestinal bacteria may be involved in gastrointestinal inflammation and promote the formation of abnormal protein aggregates in the intestine. Oral administration of the bacterial amyloid protein, curli, increases the levels of inflammatory markers and the aggregation of α-synuclein in the rat brain^26^. The intraperitoneal administration of LPS, which is a cell wall component of gram-negative bacteria, such as *Escherichia coli* and *Salmonella* spp, increases intestinal permeability and causes the accumulation of phosphorylated α-synuclein fibrils in the intestinal mucosa and dorsal vagal nuclei^27^. LPS also generates a self-renewable and structurally distinct strain of α-synuclein fibrils in vitro^28^.

PD is a multifactorial disease which is considerably influenced by environmental factors, and inheritance accounts for less than 10% of PD patients. Environmental exposure to herbicides and pesticides increases the risk of developing PD^29^. The inhibition of mitochondrial function or the induction of oxidative stress is mainly attributed to the toxicity of herbicides and pesticides^30^. Studies in mice have revealed that the pesticide rotenone, which inhibits mitochondrial electron transport complex I, causes the abnormal aggregation of α-synuclein fibrils in the ENS and subsequently in the substantia nigra pars compacta^31,32^. Vagotomy prevents the rotenone-mediated retrograde propagation of α-synuclein fibrils to the dorsal motor nucleus of the vagal nerve^33^.

## Pathology of Lewy bodies in the gastrointestinal tract

The presence of Lewy bodies in the gastrointestinal plexus was first reported by Qualman et al. in 1984^34^. Subsequently, Lewy bodies have been found in the vasoactive intestinal peptide-positive plexus but not in the tyrosine hydroxylase-positive plexus, which are both present in the myenteric plexus (Auerbach’s plexus)^35^. Some enteroendocrine cells (EECs), named neuropods, directly synapse to the vagal afferents^36^, which have a subsequent synaptic pathway to the substantia nigra and striatum^37^. In addition to the ENS, EECs also express α-synuclein^38^, which raises the possibility that α-synuclein fibrils originate in EECs and propagate to Auerbach’s neural plexus. Colon biopsies have revealed that Lewy bodies are already present in the intestinal perikarya and neurites at 2–5 years^39^ or up to 8 years^40^ prior to the onset of PD.

In PD, α-synuclein fibrils accumulate more frequently in the upper gastrointestinal tract than those in the lower gastrointestinal tract^41,42^. However, this observation remains controversial. First, the prevalence of α-synuclein fibrils in biopsies of the upper and lower gastrointestinal tracts remains undetermined^40^. Second, as the cells bearing Lewy bodies are vulnerable and decrease in abundance with the progression of PD, the upper gastrointestinal tract may have more viable cells with a small number of Lewy bodies. Indeed, ^11^C-donepezil positron emission tomography (PET) has revealed a sequential decrease in the parasympathetic innervations of the small intestine, colon, and kidney in PD^43,44^, although the presence of Lewy bodies in parasympathetic neurons has not been reported to the best of our knowledge. Therefore, the colon might have already lost cells with α-synuclein fibrils at the time of autopsy. Third, during an autopsy, the intestinal mucosa rapidly loses its structure, making histopathological evaluation difficult. Fourth, the intraperitoneal administration of LPS in mice induces α-synuclein fibrils more remarkably in the large intestine than that in the small intestine and increases intestinal permeability only in the large intestine^27^. Similar to the gastrointestinal tract, the abnormal accumulation of α-synuclein fibrils in the cutaneous sympathetic nerves has been found in the thoracic skin in two of 20 patients (10%), but none in the legs^45^. As the impairment of cutaneous sympathetic nerves is more remarkable in the extremities than that in the thorax with PD progression^45^, the lack of α-synuclein fibrils in the legs is likely to represent the loss of α-synuclein fibril-bearing sympathetic nerves. These observations may justify the apparent decrease in the number of α-synuclein fibrils in the lower gastrointestinal tract.

## Smoking, coffee, and gut microbiota in PD

Both genetic and environmental factors influence the development of PD, and genetic factors have higher effects on PD, whereas environmental factors have higher numbers of affected individuals, compared to the other factors^46^. Smoking has decreased PD development by ~50% in 44 retrospective and four prospective studies^47^. Twin studies have shown that the risk of PD is inversely correlated with the dose of cigarette smoking^48^. Nicotine in cigarettes may exert protective effects against PD^49^. Gut microbiota may also mediate protective effects. Smoking reinforces the barrier of the large intestine^50,51^, which is potentially achieved by the increased abundance of *Bacteroides* and *Prevotella* and decreased abundance of *Firmicutes* and *Actinobacteria*, which are induced by smoking^52,53^. In contrast, smoking enhances oncogenic MAPK/ERK signaling and impairs the gut barrier function in mice^54^.

Coffee consumption also reduces PD development by ~30%^55^ and is also negatively correlated with the prevalence of constipation^56^. The polyphenols in coffee may exert protective effects against PD^57^. Coffee activates gallbladder contraction and colonic motility, increases the abundance of *Bifidobacterium*, and decreases the abundance of *Clostridium*^58^.

## Constipation in PD

Constipation is observed in 54% of the patients with PD^59^. Constipation itself alters intestinal microbiota and increases mucosal permeability and inflammation. Patients with idiopathic constipation show increased serum antibody titers against *Staphylococcus aureus* and *E. coli*, suggesting the invasion of these bacteria across the orogastrointestinal barrier^60^. In addition, longer intestinal transit time is associated with the increased abundance of *Akkermansia muciniphila*, *Bacteroides* spp, and *Alistipes*^61^. The effects of confounding factors on gut microbiota in PD have shown that constipation increases the abundance of two genera (*Hungatella* and *Lactobacillus*) and decreases the abundance of three genera (*Faecalibacterium*, *Lachnospiraceae ND3007* group, and *Lachnospiraceae UCG-004*)^62^. Our study^63^, along with a previous study^64^, showed that the degree of constipation in PD patients correlates with a decrease in the serum levels of LPS-binding protein (LBP). Chronic invasion of LPS from the intestinal tract may potentially reduce serum LBP levels. In PD, intestinal permeability is increased, and *E. coli* and nitrotyrosine, markers for protein oxidization, are abnormally stained in the intestinal mucosa^64^.

## Gut microbiota in PD

Gut microbiota in PD has been analyzed using quantitative PCR, 16S rRNA sequencing (16S rRNA-seq), and shotgun metagenomic analysis (shotgun-seq), wherein qPCR has been performed in three studies^63,65,66^. Although qPCR can analyze a limited number of bacteria, it determines the absolute number of each bacterium, whereas 16S rRNA-seq and shotgun-seq determine the relative abundance of each bacterium. In our qPCR analysis, the sum of the absolute number of 19 representative intestinal bacteria, which account for 71.3% of the total intestinal bacteria, in PD was ~80% of that of controls^63^. This suggests that metabolites generated by the gut microbiota are likely to be reduced in PD.

The relative abundance of intestinal bacteria has been determined either using 16S rRNA-seq or shotgun-seq, and the results vary considerably among studies^67–72^. In addition, bacterial classifications and names vary considerably among reference databases. SILVA is the most commonly used taxonomic database, along with three other widely used databases. The difference in reference databases makes the direct comparison of different reports difficult. Thus, we established a method to meta-analyze non-parametric datasets and analyzed our own 16 S rRNA-seq dataset along with four other 16S rRNA-seq datasets^62^. Meta-analysis of gut microbiota in PD and controls across Japan, the United States, Finland, Russia, and Germany has shown an increased abundance of the mucin-degrading genus *Akkermansia* and decreased abundance of *Roseburia*, *Faecalibacterium*, and *Lachnospiraceae ND3007*, all of which produce short-chain fatty acids (SCFAs). In our dataset, the abundance of *Akkermansia* increased while that of *Roseburia* and *Faecalibacterium* decreased with PD progression. PICRUSt2 enables the prediction of functional effects from the 16 S rRNA-seq dataset. PICRUSt2 analysis has shown that butyrate and propionate metabolisms are altered in the gut microbiota in PD. This is consistent with a decrease in the abundance of SCFA-producing bacteria in PD. Metagenomic shotgun analyses^68,72,73^ also show a decrease in the abundance of SCFA-producing bacteria and an increase in that of *Akkermansia*, which is in accordance with 16S rRNA-seq analyses.

We^66,74^ and another group^70^ have reported longitudinal studies of the gut microbiota in PD. All three studies indicate that the gut microbiota is essentially unchanged over 2 years in both controls and PD. Our report shows that the increased abundance of *Bifidobacterium* at year 0 is associated with worsening in two years of the Universal Parkinson’s Disease Rating Scale (UPDRS) Part I, representing the non-motor experiences of daily living^66^. In contrast, Aho *et al*. reported that decreased *Prevotella* abundance at year 0 is associated with the rapid progression of PD^70^. Random forest models to differentiate PD patients who were at the same or better Hoehn & Yahr (HY) stage in 2 years (the stable group) and who moved to the advanced HY stage in 2 years (the deteriorated group) using gut microbiota at year 0 revealed that the abundance of only two SCFA-producing genera, *Fusicatenibacter* and *Faecalibacterium*, at year 0 were able to predict the progression of PD patients at HY stage 1 in 2 years with an area-under-the-curve of receiver operating characteristics (AUROC) of 0.868^74^. PD patients with low *Fusicatenibacter* and *Faecalibacterium* levels deteriorate faster than those with high levels of these bacteria, indicating that low SCFAs may accelerate the progression of PD.

## Gut microbiota in RBD

RBD is prodromal to PD, and ~90% of RBD patients develop other Lewy body diseases, predominantly PD. Non-negative matrix factorization of gut microbiota using Liger, a tool for single-cell RNA sequencing analysis, in controls, RBD, and PD revealed four enterotypes. The proportion of controls decreased in the order of enterotypes A to D, whereas the proportion of severe PD increased in the same order, indicating that patients with severe PD tend to have specific enterotype(s)^75^. *Akkermansia* abundance is significantly increased in RBD patients compared with that in controls in Japan and Germany^75,76^. In contrast, the abundance of SCFA-producing genera *Faecalibacterium*, *Roseburia*, and *Lachnospiraceae ND3007* group, all of which are decreased in PD, were not decreased in RBD. A low abundance of SCFA-producing bacteria may accelerate the transition of α-synuclein pathologies from RBD to PD.

## Gut microbiota in dementia with Lewy bodies (DLB)

PD patients develop motor symptoms at first, and later develop dementia, which is called PD dementia (PDD), in advanced stages. In contrast, the onset of dementia in dementia with Lewy bodies (DLB) is before or less than one year after the onset of motor symptoms^77^. DLB is characterized by visual hallucinations, fluctuating cognitive impairment, sleep disturbance, movement disorders (Parkinsonism), and autonomic dysfunctions^77,78^. The clinical symptoms, cognitive profiles, and brain pathologies are similar between PDD and DLB^79^. The factors differentiating PDD and DLB remain unknown, and the gut microbiota is a potentially responsible factor.

Our recent study has shown that the abundance of SCFA-producing bacteria is decreased, and that of *Akkermansia* is increased in DLB^80^, as has been observed in PD^62^. In addition, the abundance of *Ruminococcus torques* and *Collinsella* is increased in DLB, but not in PD. The overall gut microbiota profiles are similar in patients with DLB and PD at HY stages 3 and 4 (HY3&4), which represent moderate to severe cases of PD. A random forest model to differentiate DLB from HY3&4 shows that high *Ruminococcus torques*, high *Collinsella*, and low *Bifidobacterium* abundances are predictive of DLB with an AUROC of 0.825. Low *Bifidobacterium* abundance is also observed in Alzheimer’s disease^81,82^, and supplementation with *Bifidobacterium* improves Alzheimer’s disease, presumably by inducing the brain-derived neurotrophic factor^83^. The increased abundance of *Ruminococcus torques* and *Collinsella* are likely to cause abnormal intestinal permeability^84,85^. In addition, *Ruminococcus torques* and *Collinsella* are major secondary bile acid-producing bacteria^86^. Indeed, the measurement of fecal secondary bile acids has revealed that the production of ursodeoxycholate (UDCA), a major secondary bile acid, is increased in DLB^80^. The anti-inflammatory^87^ and antioxidant effects^88^ of UDCA have been previously reported. The effects of UDCA on PD have also been reported^89,90^. Although not directly related to Lewy body diseases, countries where healthy people have a high abundance of *Collinsella*, have low mortality rates due to COVID-19, which may also be attributed to the anti-inflammatory effects of UDCA^91^. Thus, the anti-inflammatory effects of *Ruminococcus torques* and *Collinsella* are likely to be protective against neuroinflammation in the substantia nigra and retards the development of motor symptoms in DLB.

## Intestinal bacterial metabolites in PD

Intestinal bacteria produce SCFA, vitamins, polyamines, secondary bile acids, branched-chain amino acids, trimethylamine N-oxide, tryptophan, and indole derivatives^91,92^. Shotgun metagenomic analyses have shown that biotin metabolism, glycan degradation, and the biosynthesis of phenylalanine, tyrosine, and tryptophan are decreased in PD^68,73^. High fecal concentrations of branched-chain and aromatic amino acids in PD patients are associated with an increased abundance of *Alistipes*, *Rikenellaceae_RC9_gut_group*, *Bifidobacterium*, and *Parabacteroides*, as well as a decreased abundance of *Faecalibacterium*^93^. A similar analysis has shown high levels of cadaverine, ethanolamine, hydroxypropionic acid, isoleucine, leucine, and phenylalanine in PD^94^. In contrast, glutamic, pyroglutamic, and succinic acid levels are significantly reduced in PD patients. The authors speculated that the increased intestinal metabolite levels in PD may induce oxidative stress and promote inflammatory responses and α-synuclein aggregation in ENS^95^.

In contrast to intestinal amino acids, intestinal SCFAs (acetate, propionate, and butyrate) are decreased in PD across studies^65,96–98^. Since butyrate is involved in the formation of the mucus layer in the intestinal epithelium, the intestinal mucosa may not be sufficiently formed in PD. In IBD, butyrate administration prevents the reduction of tight junction proteins and inhibits activation of the NF-κB signaling pathway by binding to GPR109A^99^. Instead, propionate is metabolized in the liver and is therefore present at low concentrations in peripheral circulation^100^. Acetic acid is the most abundant SCFA in blood. Furthermore, acetic acid can cross the blood–brain barrier and reduce appetite via central homeostatic mechanisms^101^. Acetic and propionic acids in the gut stimulate GPR41 and GPR43 to release both PYY and GLP-1, affecting satiety and intestinal transit^102^. Butyric acid exerts its anti-inflammatory effect by binding to GPR41, GPR43, and GPR109A, as well as by inhibiting histone deacetylase, the mechanism of which has been elaborated further in this review. Furthermore, propionate is converted to glucose by the gluconeogenic pathway in the intestine, which causes satiety and reduces hepatic glucose production^103,104^. The involvement of SCFA in PD pathology is addressed in the next section.

## The causal association of gut microbiota with PD

**Fig. 1:**
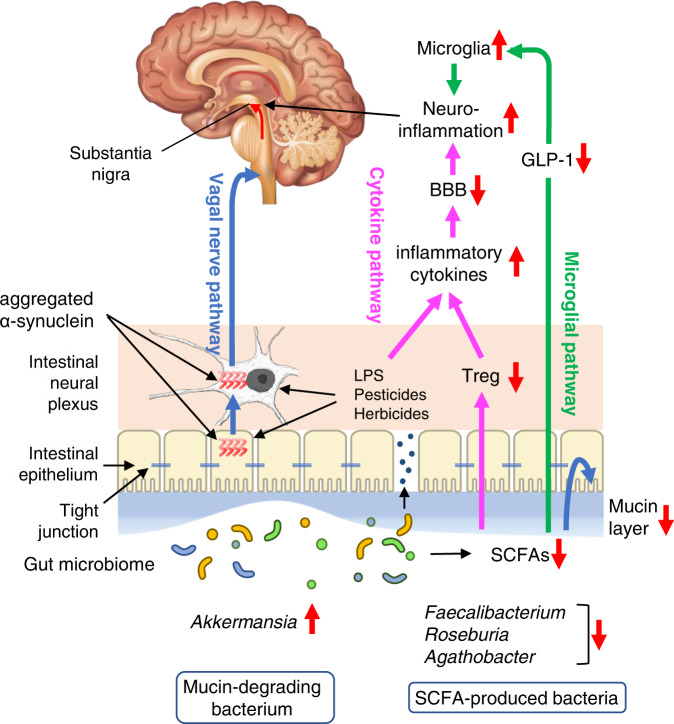
Triple-hit hypothesis of gut microbiota in Parkinson’s disease (PD). (Blue pathway) First, *Akkermansia*, which is increased in PD, degrades the intestinal mucus layer and increases intestinal permeability when dietary fibers are deficient. Thinning of the intestinal mucus layer allows toxic substances such as LPS and pesticides/herbicides to pass through the mucosal barrier, which triggers inflammation and oxidative stress in the intestine. Then, α-synuclein generated by the enteroendocrine cells and the intestinal neural plexus form insoluble fibrils. Abnormally aggregated α-synuclein fibrils ascend the vagal nerve and accumulate in the dorsal nucleus of the vagal nerve (the vagal nerve pathway). (Pink pathway) Second, *Faecalibacterium*, *Roseburia*, and *Agathobacter*, which are reduced in PD, are butyrate-producing bacteria. Butyrate is an energy source for colonic mucosal cells and is important for the maintenance of the intestinal epithelium and intestinal mucosa. Lack of SCFAs also causes thinning of the mucosal layer, and accelerates the vagal nerve pathway. SCFA also promotes the expression of Foxp3 (not shown) and the differentiation of naive T cells into regulatory T cells (Treg) by inhibiting histone deacetylases. LPS, pesticides, and herbicides additionally increase inflammatory cytokines. Peripheral inflammatory cytokines pass through the damaged blood–brain barrier (BBB) and trigger neuroinflammation. (Green pathway) Third, the lack of SCFAs also aggravates microglial activation, likely through reducing GLP-1, but the detailed mechanisms remain undetermined. (Mechanisms in DLB, not shown) Increased *Collinsella* and *Ruminococcus torques* in DLB, but not in PD, may mitigate neuroinflammation in the substantia nigra, possibly by producing secondary bile acids like ursodeoxycholate.

Based on the observations presented above, the following hypothesis was proposed for the gut–brain axis in PD (Fig. 1).

First, *Akkermansia*, whose abundance is increased in PD, degrades the intestinal mucus layer and increases intestinal permeability when dietary fiber is deficient^105,106^. *Faecalibacterium* and *Roseburia*, which are decreased in PD, produce butyrate, which is an energy source for colonic mucosal cells and is important to maintain the intestinal epithelium. Alterations in these bacteria may lead to thinning of the intestinal mucosa in PD and allow inflammatory substances such as LPS and even *E. coli* to pass through the mucosal layer^64^. Similarly, the abundance of *Bacteroides* and *Verrucomicrobia* are positively correlated with serum inflammatory cytokine levels in PD^107^. Furthermore, increased intestinal permeability also allows the enteric neural plexus to be exposed to toxins, such as pesticides and herbicides. Epidemiological studies have repeatedly demonstrated positive associations between pesticides/herbicides and PD^108^. Normal α-synuclein proteins generated in EECs and ENS are thus exposed to LPS, *E. coli*, and pesticides/herbicides, leading to the formation of abnormal α-synuclein fibrils, which may ascend the vagal nerve and accumulate in its dorsal nucleus.

In addition to PD, *Akkermansia* abundance also increases in multiple sclerosis^109^ and amyotrophic lateral sclerosis^110^. In contrast, its abundance is decreased in obesity, type 2 diabetes mellitus, autism, atopy, and IBD^111^. The paucity of *Akkermansia* in IBD may be accounted for by inflammatory thinning of the intestinal mucus layer. A high-fat diet decreases the intestinal mucus layer, and *Akkermansia* somehow rescues mucus layer thickness^112^. Increased *Akkermansia* levels may compensate for the thinned mucus layer triggered by a high-fat diet in obesity and diabetes. In contrast, a normal diet preserves the mucus layer in PD; however, an excessive abundance of *Akkermansia* may degrade the mucus layer.

Second, butyrate promotes the gene expression of *FOXP3*, and differentiates naive T cells into regulatory T (Treg) cells by inhibiting histone deacetylases^113,114^. Butyrate also acts on a G-protein-coupled receptor, GPR109a, expressed on dendritic cells and macrophages, and induces the differentiation of Treg cells and IL-10-producing T cells, leading to the expansion of Treg cells^115^. Butyrate also acts on GPR109a in the colonic epithelium and induces the production of IL-18, which also suppresses inflammation^115^. As a result, pro-inflammatory cytokine levels are elevated, and anti-inflammatory cytokines levels are suppressed in PD. The role of neuroinflammation in PD has been well-studied and reviewed^18^. For example, nonsteroidal anti-inflammatory drugs (NSAIDs) decrease the risk of PD^116,117^. Interestingly, ibuprofen, but not acetaminophen or aspirin, reduces the risk of PD by 35%^118^. Thus, a reduction in the abundance of butyrate-producing bacteria in PD may enhance neurodegeneration by failing to suppress neuroinflammation. In contrast to the protective effects of SCFAs against neuroinflammation in PD, the reduction in intestinal SCFAs owing to the depletion of gut microbiota by sanitization^119^ and antibiotics^120^ ameliorates motor deficits in rodent models of PD, whereas supplementation with SCFAs worsens them^119^. One possible reason that administration of SCFA worsened motor symptoms in a germ-free mouse model of PD^119^ was that microglia in germ-free mice was likely to be immature, because microglia requires SCFA to maturate and immature microglia cannot respond to endotoxin^121^. Administered SCFA was likely to have induced the maturation of microglia, which resulted in neuroinflammation and worsening of PD. In contrast to the germ-free mouse model of PD^119^, oral administration of butyrate protected the expression of α-synuclein in the colon and the substantia nigra, and prevented the loss of tyrosine hydroxylase-positive neurons in a rotenone-induced mouse model of PD^122^. Similarly, inulin, prebiotics yielding SCFA, mitigated microglial activation in mice^123^. More importantly, in PD patients, prebiotics yielding intestinal SCFA increased fecal and plasma SCFA levels, and decreased serum markers for intestinal and neuronal inflammations^124^.

Normal dopaminergic neurons, which are affected in Lewy body diseases, have multiple-branched, unmyelinated, and elongated neurites^125^. Energetic/metabolic burdens and subsequent oxidative stress are predicted to be high in neurons with multiple-branched neurites^126^. Thus, neurons in multiple brain regions are vulnerable to chronic neuroinflammation, and α-synuclein-expressing neurons may accumulate abnormal aggregates. This mechanism is likely to account for dopaminergic cell death in PD caused by decreased abundance of SCFA-producing bacteria. In contrast, in RBD, where *Akkermansia* abundance is increased but SCFA-producing bacterial abundance is not decreased, α-synuclein fibrils are formed in EECs and ENS and ascend the vagal nerve, but the lack of neuroinflammation is likely to suppress or delay the development of PD. Similarly, in DLB, neuroinflammation due to the lack of SCFAs is mitigated in the substantia nigra but not in the cortex by secondary bile acids generated by the increased abundance of *Ruminococcus torques* and *Collinsella*^80^. Indeed, intraperitoneal injection of LPS causes P2Y6 receptor-mediated activation of microglia and inflammatory neuronal loss in the substantia nigra but not in the cortex or hippocampus^127^. Thus, the mitigation of neuroinflammation is likely to be effective in the substantia nigra, but not in the cerebral cortex, where neuroinflammation may not critically aggravate neuronal cell death. DLB generally develops at the age of above 65 years^128^, and its average is higher than that of PD^80,128^. The lack of PD symptoms in older patients with DLB is consistent with the notion that dopaminergic neuronal cell death due to neuroinflammation at the substantia nigra is not critical in DLB. Similarly, the average age of RBD development is higher than^75^ or similar to^129^ that of PD. As stated above, the gut microbiota remains essentially unchanged over 2 years in both controls and PD^66,70,74^, and is independent of PD progression^74^. Therefore, the gut microbiota is likely to be a critical determinant of the nature and progression of brain pathology in patients with Lewy body diseases.

Early interventions for gut microbiota and their metabolites may potentially delay or mitigate the development and progression of non-motor, motor, and cognitive symptoms in Lewy body diseases.

### Reporting summary

Further information on research design is available in the Nature Research Reporting Summary linked to this article.

## Supplementary information

Reporting Summary

## Data Availability

No datasets were generated or analyzed in this article.
